# Comparing Commercial Metal-Coated AFM Tips and Home-Made Bulk Gold Tips for Tip-Enhanced Raman Spectroscopy of Polymer Functionalized Multiwalled Carbon Nanotubes

**DOI:** 10.3390/nano12030451

**Published:** 2022-01-28

**Authors:** Antonino Foti, Suriya Venkatesan, Bérengère Lebental, Gaël Zucchi, Razvigor Ossikovski

**Affiliations:** 1 CNR—IPCF, Istituto per I Processi Chimico-Fisici, Viale F. Stagno d’Alcontres 37, 98158 Messina, Italy; 2 LPICM, CNRS, Ecole Polytechnique, Institut Polytechnique de Paris, Route de Saclay, 91128 Palaiseau, France; suriya.venkatesan@dlr.de (S.V.); berengere.lebental@univ-eiffel.fr (B.L.); gael.zucchi@polytechnique.edu (G.Z.); 3 COSYS-LISIS, Université Gustave Eiffel, IFSTTAR, 77454 Marne-la-Vallée, France

**Keywords:** plasmonic nanotips, TERS imaging, MWCNT, polymer functionalization, carbon nanocomposites

## Abstract

Tip-enhanced Raman spectroscopy (TERS) combines the high specificity and sensitivity of plasmon-enhanced Raman spectroscopy with the high spatial resolution of scanning probe microscopy. TERS has gained a lot of attention from many nanoscience fields, since this technique can provide chemical and structural information of surfaces and interfaces with nanometric spatial resolution. Multiwalled carbon nanotubes (MWCNTs) are very versatile nanostructures that can be dispersed in organic solvents or polymeric matrices, giving rise to new nanocomposite materials, showing improved mechanical, electrical and thermal properties. Moreover, MWCNTs can be easily functionalized with polymers in order to be employed as specific chemical sensors. In this context, TERS is strategic, since it can provide useful information on the cooperation of the two components at the nanoscale for the optimization of the macroscopic properties of the hybrid material. Nevertheless, efficient TERS characterization relies on the geometrical features and material composition of the plasmonic tip used. In this work, after comparing the TERS performance of commercial Ag coated nanotips and home-made bulk Au tips on bare MWCNTs, we show how TERS can be exploited for characterizing MWCNTs mixed with conjugated fluorene copolymers, thus contributing to the understanding of the polymer/CNT interaction process at the local scale.

## 1. Introduction

Plasmonic nanotips can focus and amplify optical fields into nanosized volumes, in the so-called near-field region [1,2], far below the diffraction limit. This is possible owing to the resonant excitation of the localized surface plasmon (LSP) of the nanostructured tip (or probe), which acts as a nanoantenna that strongly enhances the electromagnetic field at the tip apex, turning it into a highly efficient light nanosource, i.e., a hot-spot [2,3]. This is the basic principle behind tip-enhanced Raman spectroscopy (TERS) [4,5], where a nanotip made of gold or silver squeezes the far-field components of a laser beam into the near-field region and vice versa. This allows plasmon-enhanced Raman measurements at the nanoscale and even down to the single-molecule level [6,7]. When a TERS tip is integrated into a scanning probe microscope (SPM), such as scanning tunnelling microscopy (STM), atomic force microscopy (AFM) or shear-force microscopy (ShFM), the hot-spot position can be precisely controlled, giving rise to a powerful near-field optical technique, which adds fast chemical imaging of surfaces and interfaces [4,8] to the conventional SPM morphological characterization, with spatial resolution reaching the nanometric size or even the atomic scale in ultra-high vacuum (UHV) conditions [7,9].

From a practical point of view, the substrate on which the analyte lies plays an active role in TERS measurements by directly influencing the value of the field enhancement and the ultimate chemical sensitivity [4,8,10]. Optimal TERS performance is obtained in the so-called gap-mode configuration where the nanotip is in close proximity (less than 1–2 nm) to a plasmonic substrate, so that TERS enhancement takes advantage of an additional boost (of up to 2–3 orders of magnitude) if compared to a non-coupled configuration. This is due to the occurrence of a strong cavity mode, which also induces a red shift in the tip LSP resonance (LSPR) [4]. Nevertheless, the main character in a TERS measurement is the tip whose features directly influence spatial resolution, reproducibility and signal enhancement [8,11]. Both silver and gold are good material choices, providing exploitable TERS response in the visible excitation range [11]. Silver tips have a better plasmonic response but suffer from oxidation and sulfurization that induces fast quality degradation, resulting in poor TERS performance under ambient conditions [12,13]. Nevertheless, these issues can be overcome by coating the tip with a protective layer, usually made of oxides, at the cost of consequent loss in the spatial resolution due to the increase in tip dimensions [14]. On the other hand, gold tips are widely employed despite their lower signal enhancement because of their high chemical stability [11]. Depending on the SPM technique used, TERS tips can present different morphologies. AFM−TERS tips are usually fabricated by coating commercial AFM silicon or silicon nitride probes with plasmonic metals using evaporation techniques or electrodeposition [15,16]. Evaporation parameters play a fundamental role in the optimal fabrication of TERS tips due to the fact that the quality of adhesion and the roughness of the evaporated film can be crucial for the final enhancement of the scattered signal [17]. STM−TERS tips are usually fabricated by means of chemical [18] or electrochemical [19,20,21] etching process from metal wires, obtaining very sharp bulk tips. Etched-wire bulk TERS tips are generally preferable to metal-coated ones, since they typically feature lower tip apex radii (<10 nm against 20–30 nm) and higher field-enhancement levels [11]. Bulk plasmonic tips are usually employed in ShFM−TERS, since they can be glued to the arm of a quartz tuning fork [11]. If the tip is glued with its axis perpendicular to the arm of the tuning fork, then the feedback system is based on normal forces (NF) rather than on shear forces [22]. At the beginning of TERS, coated AFM tips and bulk STM tips with comparable apex sizes usually showed huge differences in TERS enhancement [23], with AFM–TERS tips procuring TERS signals with very poor reproducibility and enhancement levels almost two orders of magnitude lower compared to STM tips, which can routinely provide enhancement factors (EF) of about 10^5^–10^6^ [23]. However, over the past few years AFM tip metallization techniques have dramatically improved, providing competitive EF values in the range 10^3^–10^5^ [15,16,17,24]. At present, several companies are commercializing AFM–TERS tips [25,26,27], providing high enhancement factors (up to 10^6^), spatial resolution (<15 nm) and reproducibility [26]. These new-generation AFM–TERS probes offer novel efficient solutions for the chemical characterization at the nanoscale of a wide range of samples. In fact, bulk plasmonic tips are mainly employed in STM and, therefore, on conductive samples or in tuning-fork-based SPM (TF-SPM), which has a slower feedback response compared to AFM.

The huge effort in technological improvements has been driven by the high appeal TERS has quickly gained in many application fields, both in material and life science [28]. In the latter, the ability to study biomolecule structure and conformation at the nanoscale opens new routes for the understanding of the biological processes and functionalities in living organisms, permitting, for example, to distinguish between toxic and non-toxic configurations [29,30]. Other applications of TERS in bioscience include the study of biological systems, such as RNA, DNA, proteins, collagen, bacteria and viruses [28,31,32,33,34], as well as the in vivo characterization of the protein uptake by yeast cell envelopes [35]. Very recently, the implementation of new detection schemes has made it possible to measure TERS in liquid environment with the development of the first electrochemical TERS setup [36], paving the way for the study and the control of chemical and/or physical processes at a local level [37,38]. Nevertheless, a major scientific field for the TERS technique is still represented by material science. TERS has been employed in the characterization of ferroelectric nanodomains in crystals [39], mineral composition at the nanoscale [22], inorganic nanowires [40], self-assembled monolayers of molecules grafted on gold surfaces [41,42,43,44,45], carbon-based materials such as carbon nanotubes (CNTs) [46,47,48,49,50] or graphene [51,52,53], as well as in the non-destructive in situ chemical identification of pigments in artworks [54]. Last but not least, TERS imaging on carboxyl-modified graphene oxide flakes have allowed the imaging of the structural composition of the sample surface with a resolution of about 10 nm, providing the means for a systematic study of point defects and functional groups present on the material [55].

CNTs are very versatile nanomaterials that are applied in many industrial fields. For instance, single-walled CNTs (SWCNTs) are regularly integrated into high-performance electronic devices or solar cells [56,57]. Moreover, multiwalled CNTs (MWCNTs), consisting of two or more concentric CNTs, are widely employed in many industrial applications owing to their lower synthesis cost and large volume manufacturability [58]. MWCNTs can be dispersed within polymer matrices from solutions and used as nanofillers to improve the mechanical, thermal and electrical properties of polymers [59,60,61,62], giving rise to new nanocomposite materials [63,64]. Moreover, CNTs, functionalized by non-covalent interactions with amphiphilic entities, such as conjugated polymers, have been proposed as active materials in sensors [65,66,67], in which the polymer, while stabilizing the CNT dispersion, can specifically interact with analytes [66,68]. In order to fully understand the mechanisms behind the CNT/polymer cooperation, the study of local interactions between the nanofiller and the matrix occurring at the interfaces is crucial. By providing spectroscopic information at the nanoscale, TERS represents an ideal technique in fulfilling this task. In this context, Suzuki et al., exploited TERS for the characterization of styrene–butadiene rubber-MWCNT nanocomposite [69], highlighting that the local order of the polymer chains at the interface is dependent on the π−π non-covalent interaction between the phenyl rings of the polymer and the sp^2^/sp^3^ carbon atoms of the MWCNT walls [65,70]. The same group also carried out TERS measurements on SWCNTs/polystyrene nanocomposites, characterizing at the local level the mechanical stress introduced by the polymer compression on the SWCNTs [71].

In the first, instrumental part of this work, we report on a quantitative comparison of the TERS performance of commercial Ag coated AFM–TERS tips [26] and home-made bulk Au tips [45] on bare MWCNTs. In the second, application-oriented part, we exploit the TERS technique to characterize MWCNTs interacting with conjugated fluorene copolymers, providing new insight into the polymer/CNT functionalization process.

## 2. Materials and Methods

### 2.1. Polymer-Functionalized MWCNTs Synthesis

MWCNTs (NC3100 from Nanocyl, Sambreville, Belgium) were dispersed in 1,2-dichlorobenzene (DCB) at 0.02 wt.%. The solution was sonicated using ultrasonic probe (Bioblock Scientific, Illkirch, France—VibraCell 75043) at 150 W/20 °C/20 kHz/20% Ampl for 30 min with 15 s interval. The dispersion was centrifuged at 8000× *g* for 30 min, followed by second centrifugation at 10,000× *g* for 4 h (Thermo Scientific, Waltham, MA, USA—Megafuge 8). In both steps, the pellet was discarded and the suspension (CNT ink) was collected. The final concentration of the prepared CNT ink was found to be 0.05 mg per mL, which was 20% of the initial CNTs taken [72]. Polymer solution was obtained through solubilizing Poly(1,1′-((9′,9′-dihexyl-9H,9′H-[2,2′-bifluorene]-9,9-diyl)bis(4,1-phenylene))bis(3-phenylurea)) (FFUR-14) [73] in DCB. Subsequently, it was stirred at 3000 rpm for 48 h, followed by filtration through a filter, with pores of 0.45 µm, in order to remove the undissolved polymer aggregates. Finally, CNT ink was mixed with the polymer solution and sonicated for 5 min in an ultrasonic bath. The prepared nanohybrid ink was aged for 24 h before usage in order to optimize the formation of polymer-functionalized CNTs (f-CNTs) [70,73]. Finally, the mixed solution was centrifuged in order to separate the f-CNTs from free or loosely bound polymers.

### 2.2. TERS Substrate Preparation

Flat crystalline (111)-gold coated substrates (1.1 × 1.1 cm^2^; from Arrandee, Werther, Germany) were used for our analysis. The substrates use a borosilicate glass base of 0.7 mm thickness on top of which a gold layer (250 nm ± 50 nm) is deposited. Flame annealing was performed on them prior to their use in order to enlarge the crystalline domains. Subsequently, the gold substrates were cleaned using DCB and immersed for 8 h in the f-CNT ink solution, with a polymer-to-CNT ratio of 1:2 (*w*/*w*). After the dip casting procedure, the substrates were dried at 80 °C for 10 min and gently washed in DCB and in distilled water in order to remove the excess material, thus avoiding big deposits of CNTs on the substrate surface.

### 2.3. TERS Setup

**Figure 1:**
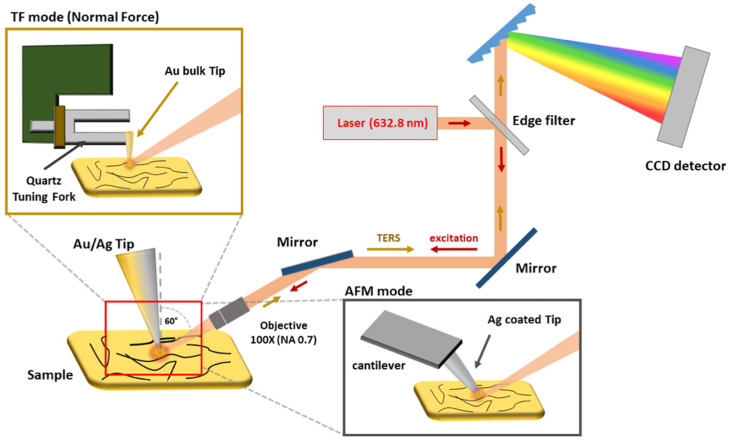
TERS setup. Schematics of the experimental setup showing the SPM system that can be operated either in AFM mode or in TF mode based on normal force interaction. A red laser (*λ_exc_* = 632.8 nm) is coupled via a 100× lens (NA = 0.7) onto the apex of the plasmonic tip at an angle of 60°. The TERS signal is collected in a backscattering configuration.

TERS experiments were carried out at ambient conditions with a confocal Raman spectrometer (LabRam HR800—Horiba Scientific, Palaiseau, France) equipped with a He-Ne laser (emitting at 632.8 nm) and coupled with a SmartSPM-1000 system (AIST) in a side (or off-axis) configuration. The laser beam is focused onto the tip apex using a 100× long working distance lens (NA = 0.7), forming an angle of 60 degrees with the sample normal (Figure 1). On the other hand, TERS tip axis can present a variable angle (ranging from almost 0 to about 15 degrees) with respect to the sample normal axis from the side opposite the incoming laser beam. The scattered signal is collected through the same path in a backscattering configuration and dispersed by a 600 lines/mm diffraction grating on a CCD detector (Synapse—Horiba Scientific). The AIST system was operated both in AFM–TERS, as well as in TF–TERS mode, in which the tuning fork is parallel to the surface, and a bulk gold tip is glued perpendicularly to the lower arm. In both modes, the tip is less than 1–2 nm above the sample surface. AFM–TERS silver-coated tips (radius of curvature 30–40 nm) were purchased from HORIBA Scientific [26] (see Supplementary Material Figure S1), while the gold tips were fabricated from Au wire of 250 µm diameter (Goodfellow AU005140, high purity 99.99%, temper hard) by electrochemical etching, according to the method described in reference [19], featuring tip apexes with radii of curvature in the range 20–50 nm, as showed elsewhere [45]. Therefore, an average radius of curvature (*R*) of 35 nm ± 10 nm can be assumed for both kinds of tips. Laser power was set either at 1.1 mW or at 450 µW, and acquisition time was kept in the 0.5–5 s range in order to avoid polymer degradation and to minimize the thermal drift.

## 3. Results and Discussion

### 3.1. Morphological Characterization of MWCNTs

**Figure 2:**
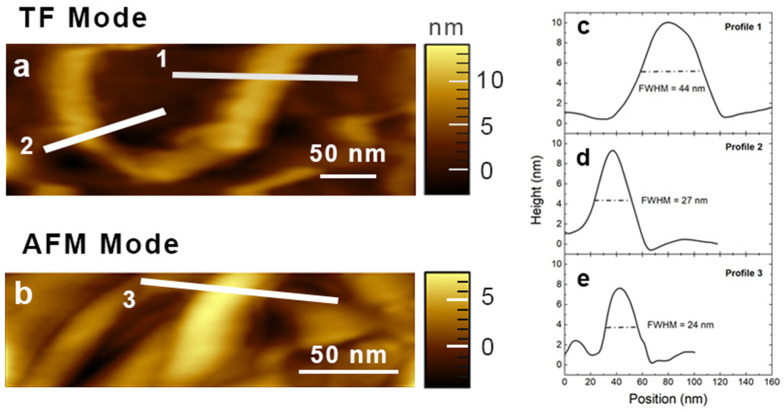
Topographic characterization. Topographic imaging of MWCNTs on gold substrate obtained in (**a**) TF mode and in (**b**) AFM mode. (**c**–**e**) Line profiles relative to Section 1, Section 2 and Section 3, respectively.

In addition to plasmonic amplification, TERS tips are employed simultaneously in morphological characterization of nanostructured samples where the tip apex dimension determines the resolution of the imaging. As a consequence, the spatial resolution of metallic/metallized tips is lower compared to the one obtained with classical silicon tips featuring R≈1 nm. Figure 2 compares the performance of commercial metallized AFM tips with bulk metal TF tips employed in the morphological characterization of MWCNTs. In both TF (Figure 2a) and AFM (Figure 2b) modes, we can distinguish the MWCNTs lying on the substrate and showing height values in the range 7.5–10 nm, in agreement with the technical data provided by the CNT supplier (9.5 nm). Profile 1 in Figure 2c likely highlights the presence of at least two MWCNTs arranged in a side-to-side configuration. Profile 2 (Figure 2d) and profile 3 (Figure 2e) are obtained in TF mode and AFM mode, respectively (from different sample regions); they both feature an apparent width of about 25 nm, which suggests they are very likely due to single MWCNTs. These results confirm that our home-made Au tips (working in TF mode) and commercial Ag tips (working in AFM mode) feature quite similar apex dimensions, since they provide comparable topographic images.

### 3.2. TERS Enhancement of MWCNTs

**Figure 3:**
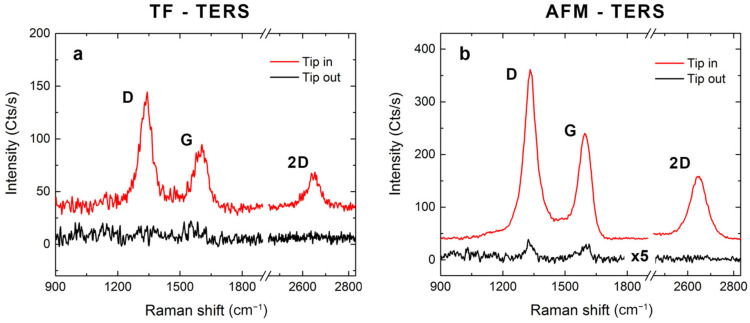
TERS enhancement. Raman signal of MWCNTs on gold substrate observed with (red line) and without (black line) the TERS tip in close proximity (1–2 nm) to the sample surface and acquired in (**a**) TF mode and (**b**) AFM mode. Experimental conditions: (**a**) *p* = 1 mW; (**b**) *p* = 0.45 mW. Data are offset for clarity.

In order to compare the TERS performances of the two tips, we need to estimate the TERS enhancement factor (*EF*) in the first place. This parameter is directly related to the contrast (C) given by [23]:

(1) $$C=\frac{I_{Tip-in}}{I_{Tip-out}}$$

where $I_{Tip-in}$ and $I_{Tip-out}$ is the scattered signal, collected with and without the tip inside the near-field region of the sample, respectively. Notably, $I_{Tip-out}$ represents the far-field intensity ($I_{FF}$), while $I_{Tip-in}$ is the sum of the near-field intensity ($I_{NF}$) and $I_{FF}$. Since the sample considered here is a very thin film of nanostructures, the *EF* can be calculated from the following relation [21,23]:

(2) $$EF=\left(C-1\right)\frac{A_{FF}}{A_{NF}}$$

in which $A_{FF}$ is the scattering area corresponding to $I_{FF}$, i.e., the area of the laser spot on the sample surface (in a side configuration, it is an ellipse rather than a circle). Next, $A_{NF}$ is the near-field area sensing the field enhancement due to LSP excitation and is usually approximated by the tip apex dimension ($A_{NF}\approx \pi R^{2}$) [10,21]. In Figure 3a, the contrast measurements obtained in the TF mode are shown. When the Au bulk tip is at working distance (i.e., at ≤1–2 nm from the sample surface) we can clearly observe the typical Raman spectrum of MWCNTs, featuring the D band (~1330 cm^−1^), the G band (~1590 cm^−1^) and the 2D band (~2650 cm^−1^) [74,75]. Whereas the G band is due to C–C stretching mode, the D peak originates from vibrational modes activated by structural defects in the carbon hexagonal lattice [76]. However, in the case of MWCNTs, the D band also benefits from the contribution coming from the interwall interactions all over the nanostructure [77]. Finally, the 2D band is related to the electronic properties of the carbon nanostructures. It is the overtone of the D band and, being a two-phonon process, it can be activated even in the absence of defects [74,75]. When the tip is retracted from the surface (50 nm away), the amplified field does not have any influence on the sample and thus, only a flat signal is recorded. This means we can estimate the lower limit of the TERS EF by considering the noise level of the spectrum as $I_{FF}$ [21]. Therefore, taking an average tip radius of 35 nm, we obtain $EF_{D}>1.7\times 10^{4}$, $EF_{G}>8.5\times 10^{3}$ and $EF_{2D}>5.7\times 10^{3}$ for the D, G and 2D bands, respectively. On the other hand, in the case of AFM–TERS, the far-field spectrum likewise presents the peaks characteristic of the CNTs (black line in Figure 3b), which allows a more precise estimation of the enhancement for the D band ($EF_{D}\approx 5.4\times 10^{4}$) and the G band ($EF_{G}\approx 4.9\times 10^{4}$). For the 2D band, we have $EF_{2D}>8.8\times 10^{4}$, since no peak could be observed in this spectral range without the plasmonic tip in the near-field region. This confirms that the amplification process exhibits spectral dependence, with better efficiency at higher frequencies. In both TERS modes, the variability in the order of magnitude of the EFs calculated on different positions of the sample is ≤10%. From these first considerations, we can reasonably conclude that coated and bulk tips provide comparable results from the point of view of field enhancement, in contrast to what was experienced in the first years of TERS tips fabrication, where coated AFM–TERS tips featured very low amplification values in comparison to bulk plasmonic tips (amounting to almost two orders of magnitude of difference) [23].

### 3.3. TERS Resolution and Structural Characterization of MWCNTs

The high appeal of the TERS technique is due to its ability to chemically resolve analytes and small objects at the nanoscale. The ultimate spatial resolution achievable by a TERS imaging system is strictly related to the plasmonic tip properties (apex radius and field enhancement value), to the ambient conditions (temperature, pressure…) and to the stability of the piezo-electric stage controlling the relative position of the probe and the sample [10,11,78]. Generally, the smaller the tip radius, the better the resolution [11]. In addition to this, it has been demonstrated very recently that TERS lateral resolution can be pushed down to the atomic level, i.e., to a size range much smaller than the tip radius (R~10 nm), which makes possible the imaging of single vibrational modes [9,79]. This outstanding result is ascribed to the presence of plasmonic picocavities formed by a plasmonic flat substrate and some atomic protrusions at the very end of the TERS tip [10,80]. This means it is possible to get a higher resolution by decreasing the tip-to-sample distance [10]. However, the stability of this approach is strongly limited by the environmental conditions of the measurements, requiring UHV setups working at low temperature for atomic TERS resolution [7,9]. Our experimental configuration, being operated at ambient conditions, is intrinsically limited in the minimum gap obtainable between the tip and the sample.

**Figure 4:**
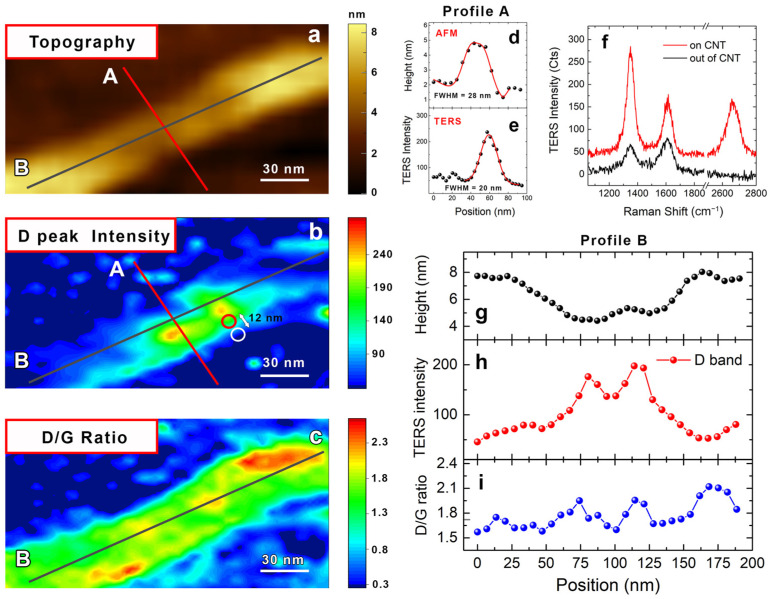
TERS characterization of MWCNTs in AFM mode. (**a**) Morphological image of a MWCNT acquired in AFM mode. TERS image of the MWCNT from panel (**a**), obtained by mapping the D band intensity (**b**) and D/G intensity ratio (**c**) (mapping step size of 5 nm). Line profile (A) in both (**d**) topographic and (**e**) D band maps corresponding to the red lines in panels (**a**,**b**), respectively. (**f**) Point spectra of the TERS map corresponding to the red circle (red curve) and the white circle (black curve) in panel (**b**). The spectra are separated by only 12 nm. Data are offset for clarity. Line profile (B) corresponding to the black line in panels (**a**–**c**) showing the height (**g**), D band intensity (**h**) and D/G ratio (**i**), respectively. Experimental conditions: *p* = 0.45 mW, *T* = 0.5 s.

In this section, we estimate the resolution achievable by our tips in AFM and TF gap-mode TERS on MWCNTs under ambient conditions. In Figure 4, we directly compare the AFM topographic image of a bare MWCNT (Figure 4a) with the nano-Raman image performed in AFM–TERS mode on the same nano-object (Figure 4b). Figure 4b shows the CNT TERS image, obtained by mapping the D peak intensity after background removal in AFM–TERS mode, using a step size of 5 nm. Since in MWCNTs the D band is also due to the interwall interactions and not only to the presence of structural defects, it can be used to image the nanostructure in its whole extension [77]. In order to estimate the spatial resolution in both AFM and TERS images, we made a line profile in a direction perpendicular to the CNT axis (profile A) in both topographic and D-band intensities (red lines in Figure 4a and Figure 4b, respectively), obtaining (after data fitting) a FWHM of 28 nm (Figure 4d) and 20 nm (Figure 4e), respectively. This means that we are able to image our sample with a resolution that is better than the one we can typically get by simple morphological analysis in the AFM mode. In addition, Figure 4f demonstrates the high signal contrast achievable in TERS imaging. Indeed, the upper spectrum clearly shows the vibrational fingerprint of the CNTs (red line—corresponding to the red circle in Figure 4b), while the lower one presents a signal only related to carbon contamination on the sample (black line—corresponding to the white circle in Figure 4b), a fact supported by the different ratios between the D and the G bands and the absence of the 2D peak in the high-frequency region [81]. Since the two spectra are taken at the distance of only 12 nm from each other, the actual resolution can be even higher. These experimental observations are, likewise, compatible with the theory, since the near-field region is confined in a volume whose linear size is given approximately by $\sqrt{Rd}$, where d is the tip-to-sample distance [10]. This expression defines the TERS spatial resolution, which, in our conditions, considering d< 1 nm and R≈35±10 nm, can be evaluated to lie approximately within 5 and 7 nm.

Very interestingly, in the central region of the CNT, the D band is extremely intense compared to the CNT extremities. This additional contribution to the Raman signal can be justified by inspecting the topographic profile and the TERS intensity along line B in Figure 4g,h, respectively. The inspection shows that the higher D-band intensity is in exact correspondence with the smaller height of the CNT. The latter is likely due to a broad structural damage of the nanostructure. In fact, any morphological depression related to the substrate can be excluded, since a line profile parallel to line B and lying outside the CNT provides an almost flat line (not shown). This means that the nanotip, working in contact mode during the TERS measurements, is at least 3 nm closer to the gold substrate in the central part with respect to the CNT extremities (Figure 4g). Therefore, the smaller plasmonic gap gives rise to a stronger field enhancement, yielding a more intense TERS response [4,8]. Finally, the additional signal of the D band cannot be ascribed to a possible excess of structural defects since, in mapping the D/G ratio, the CNT image (Figure 4c) presents different features compared to those in Figure 4b. Notably, in contrast to the D band image (Figure 4b), the D/G image is in a better correspondence with the topography (Figure 4a) because the peak ratio compensates the variability of TERS enhancement due to the modification of the tip-to-substrate distance. Moreover, Figure 4c provides an extra way to assess the homogeneity of the sample, as well as to locate potential structural defects [74,82]. In our analysis, the zone with higher density of defects is observed in correspondence with a bending of the MWCNT (top right of the image), where the probability of the presence of mechanical stress is higher, whereas, in the central part of the CNT, it is in correspondence with the smaller diameter (Figure 4h), thus confirming our initial hypothesis that the reduced CNT height in this area is due to a possible structural damage.

**Figure 5:**
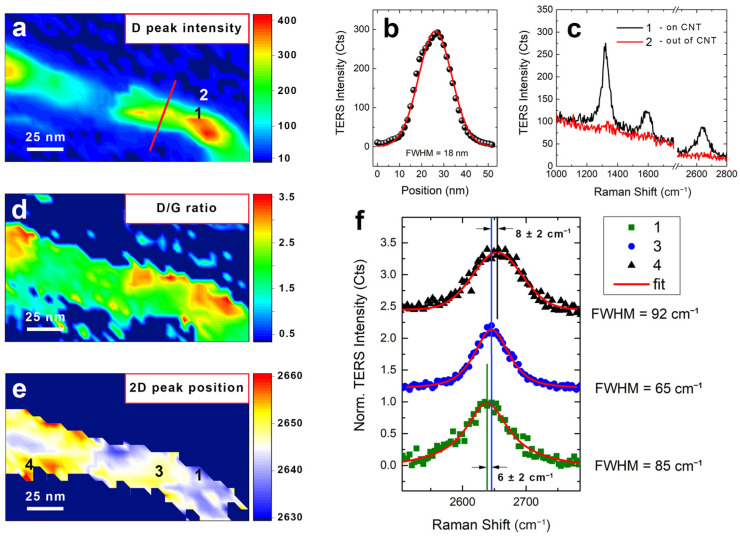
TERS characterization of MWCNTs in TF mode. TERS image of a MWCNT obtained by mapping the D band intensity (**a**) and the D/G intensity ratio (**d**) (step size of 5 nm). (**b**) Line profile of the D band intensity corresponding to the red line in panel (**a**). (**c**) Point spectra of the TERS map corresponding to positions 1 (on CNT—black curve) and 2 (out of CNT—red curve), marked in panel (**a**). The spectra are separated by only 10 nm. (**e**) TERS map of the 2D position of the MWCNT from panel (**a**). (**f**) Point spectra of the TERS map corresponding to positions 1 (green squares), 3 (blue circles) and 4 (black triangles), marked in panel (**e**). Red solid lines are Lorentzian fits. Data are offset for clarity. Experimental conditions: *p* = 1.1 mW, *T* = 0.5 s.

An analogous nano-Raman characterization was performed on the same sample employing the TF–TERS mode. Figure 5a shows the TF–TERS image of a single CNT obtained by mapping the D band peak intensity after background removal, using a step size of 5 nm, like in the AFM–TERS imaging of Figure 4. In TF–TERS mode, like in the AFM–TERS one before, the spatial resolution was estimated from a line profile featuring a FWHM of 18 nm after data fitting (Figure 5b). This value is comparable with the one achieved with coated AFM tips (Figure 4e), meaning that in TF–TERS mode, we are also able to image our sample with a resolution likely higher than the one we can typically get from a pure morphological analysis (Figure 2d). Following the same procedure used in AFM–TERS mode, we demonstrate the high contrast of the TF–TERS response in Figure 5c, where the upper spectrum clearly shows the vibrational fingerprint of the CNTs (position 1), while the lower one presents a flat signal (position 2). Position 1 and position 2 are separated by only 10 nm, suggesting that, also in this case, the ultimate resolution achievable in TF–TERS imaging can be higher. The richness of information contained in the vibrational spectrum enables a more detailed characterization of the MWCNTs in addition to simple imaging. Notably, in addition to assessing the homogeneity of the sample and the defect localization from the D/G intensity ratio TERS map (Figure 5d) [74,82], it is also possible to retrieve other structural information from the spectral position of the 2D peak whose shift can be related to the mechanical stress present in the CNT [74]. Specifically, in case of tensile deformation, the carbon bonds of the CNT lattice will be elongated, lowering the vibration energy and, thus, resulting in a downshift of the 2D peak position and a broadening of the FWHM [83]. Conversely, if the CNT endures a compressive stress, then the 2D position upshifts [84]. This is in agreement with what is observed from the 2D peak position map of our sample (Figure 5d), where the bent part of the CNT (position 1), experiencing tensile deformation, features peak position values lower than the ones corresponding to the central, unstressed portions of the tube (e.g., position 3). This observation is further supported by inspecting the TERS spectrum at position 1 (dark green squares in Figure 5f) whose 2D band downshifts by up to 6 cm^−1^ compared to the one at position 3 (blue dots in Figure 5f), which additionally presents a narrower FWHM. Moreover, the map reveals the presence of zones of compressive stress featuring a 2D peak upshift like, for example, at position 4 (black triangles in Figure 5f), characterized by a positive spectral shift of 8 cm^−1^ from the 2D peak at position 3 (blue circles).

### 3.4. TERS Characterization of Polymer-Functionalized MWCNTs

**Figure 6:**
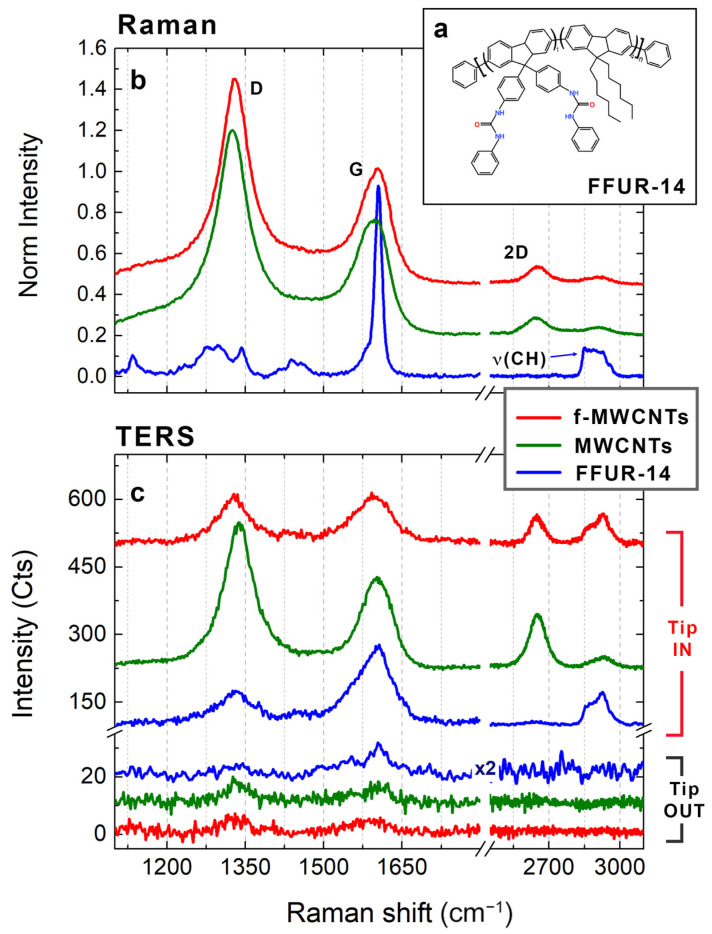
Raman vs. TERS signal of f-MWCNT. (**a**) Structural formula of the FFUR-14 polymer. (**b**) Normalized conventional Raman spectra of f-MWCNT (red curve), bare MWCNT (green curve) and FFUR-14 polymer (blue curve) deposited on a flat gold surface. Experimental conditions: *p* = 2.0 mW, *T* = 10 s. (**c**) TERS contrast measurements performed on f-MWCNT (red curve), bare MWCNT (green curve) and FFUR-14 polymer (blue curve) deposited on a flat gold surface. The top spectra (Tip IN) are obtained with the AFM–TERS tip in the near field of the sample, while the bottom ones (Tip OUT) are the corresponding signals recorded with the tip removed. Experimental conditions: *p* = 0.45 mW, *T* = 3 s. Data are offset for clarity.

The high chemical resolution mapping capability of TERS is strategic in the characterization of CNT nanocomposites. In fact, the analysis of the interfacial interactions at the nanoscale allows the retrieval of information useful for the optimization of the macroscopic properties (i.e., mechanical strength, electrical/thermal conductivity…) of the hybrid material [61,62,64,85,86]. TERS has already been employed in the study of the surface interactions between the polymer and the CNT [69,71] and has provided a new insight on the relative orientation between the polymer and the CNT [69], suggesting that the main bonding mechanism is based on the π−π non-covalent interactions between the delocalized electrons of the aromatic rings present in the polymeric chain and the π electrons of the sp^2^ carbons of the CNT lattice [69]. The polymer used for the functionalization of our MWCNT sample is the FFUR-14 (Figure 6a), whose backbone is composed by fluorene groups bearing either dihexyl units or bis urea group (one every nine fluorene/dihexyl groups) [70]. Calculations of the electronic structure of FFUR-14 show that it can efficiently bind to the CNT surface through π−π interactions, giving rise to a stable functionalized nanomaterial, with the fluorene groups either parallel or perpendicular to the CNT surface within few nanometers, with no preferential configuration [70]. The typical Raman spectrum of FFUR-14 is shown in Figure 6b (blue line). It is characterized by the typical Raman fingerprint of the aromatic fluorene group, whose strong peak at 1604 cm^−1^ is due to the in-plane mode of the main polymer chain [87,88]. An additional band related to the polymer is observable in the high-frequency region. Centered at 2900 cm^−1^, it is attributed to the symmetric and asymmetric CH stretching modes of the aliphatic chains [69]. Unfortunately, the main band of FFUR-14 lies in the same spectral region as the G band of the MWCNT, making it difficult to clearly identify the polymer within the hybrid material. In fact, the conventional far field Raman response of the f-MWCNT (red curve in Figure 6b) is nearly unchanged compared to the spectrum of the bare MWCNTs (green curve in Figure 6b). Even if the G band shape is slightly modified because of the polymer band at 1604 cm^−1^, the D/G ratio is essentially the same, and the CH stretching band of the polymer is completely drowned in the D + G peak of the MWCNTs at 2900 cm^−1^, which is a combination of the D and G bands [89]. However, a different behavior is observed when the f-MWCNT sample is characterized by the TERS technique. In particular, Figure 6c demonstrates the excellent TERS contrast obtainable in AFM–TERS mode on f-MWCNT sample (red curves), on bare MWCNTs (green curves), as well as on FFUR-14 polymer (blue curves). The three samples were deposited on gold substrates in order to benefit from the huge enhancement provided by gap-mode TERS. The detected signal is almost flat with the tip retracted for all three samples (bottom curves in Figure 6c), especially in the high-frequency region. When the tip is engaged in the feedback loop of the AFM system, a well-structured Raman fingerprint is visible in all three cases, even at very low power levels (450 µW) and only 3–5 s of integration time (top curves in Figure 6c). As evident from the comparison between the red and the green spectra in Figure 6c, the difference between bare MWCNT and f-MWCNT is revealed much more clearly in TERS rather than in conventional Raman. In fact, the f-MWCNT TERS response is characterized by a lower D/G ratio with respect to the typical value of ~1.78 relative to the conventional Raman signal of the same sample (red line in Figure 6b). Moreover, the CH stretching band at around 2900 cm^−1^ of the polymeric chain overcomes the D + G mode of MWCNTs and is visible, together with the 2D band. The shape of the high-frequency modes observed in the TERS spectrum of bare FFUR-14 on gold (top blue curve in Figure 6b) confirms the polymeric nature of the band observed in f-MWCNT TERS, providing a clear marker for the presence of FFUR-14 in our hybrid sample.

**Figure 7:**
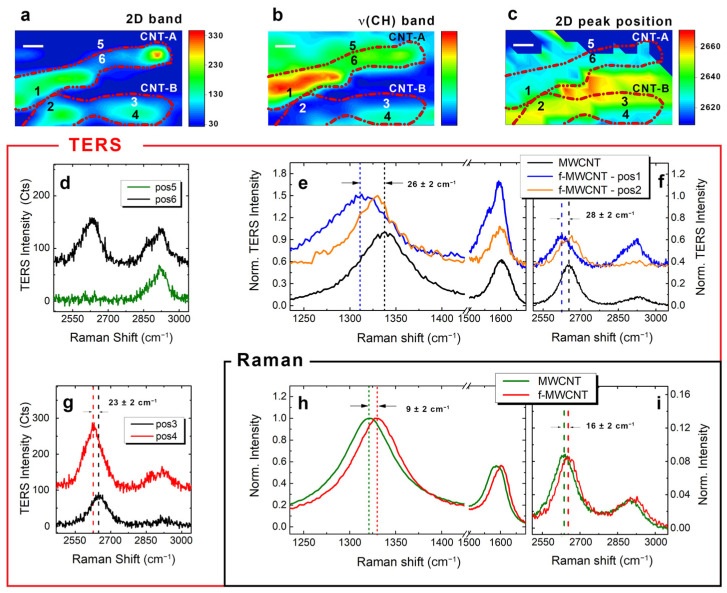
TERS characterization of f-MWCNTs in AFM mode. TERS imaging of an area of about 200 × 130 nm^2^ obtained by mapping (**a**) the 2D band of the MWCNT, (**b**) the CH stretching mode of the FFUR-14 and (**c**) the position of the 2D peak (step size of 15 nm). Red dashed lines in the three maps are the contours of the MWCNTs localized from the images in panel (**a**), while the scale bar is 25 nm. (**d**) Point spectra of the TERS map corresponding to position 5 (green curve) and 6 (black curve), marked in panels (**a**–**c**). The spectra are separated by only 15 nm. (**e**,**f**) Point spectra of the TERS map corresponding to position 1 (blue curve) and 2 (orange curve), marked in panels (**a**–**c**). (**g**) Point spectra of the TERS map corresponding to position 3 (black curve) and 4 (red curve), marked in panels (**a**–**c**). The spectra are separated by only 15 nm. (**h**,**i**) Normalized Raman spectra of bare MWCNTs (green curve) and f-MWCNTs (red curve). Raman experimental conditions: *p* = 2.0 mW, *T* = 10 s. TERS Experimental conditions: *p* = 0.45 mW, *T* = 0.5 s. Data are offset for clarity.

In Figure 7a, we present the AFM–TERS image of the f-MWCNT sample within an area of about 200 × 130 nm^2^. The image, obtained by mapping the 2D intensity of the CNT, allows us to localize two MWCNTs on the substrate (dashed red profiles). Very likely, these are two individual CNTs, since the intensity line profiles provide a FWHM of about 20 nm. Furthermore, Figure 7b shows the image of the same region, constructed by mapping the CH stretching band intensity, thus providing the distribution of the polymer within the sampled area. By superimposing the CNTs dotted profiles from Figure 7a on this map, we can clearly notice that CNT-A is fully covered by the polymer, while CNT-B is only partially covered. Other zones are characterized by the presence of polymer only (e.g., position 5—green curve in Figure 7d) and can be easily distinguished from zones with polymer and MWCNTs within only 30 nm (e.g., position 6—black curve in Figure 7d). Nevertheless, at this stage, a contribution from sonopolymers, possibly created upon sonication of the solvent (DCB), cannot be excluded [90]. Indeed, the CH stretching band is present in both the FFUR-14 and DCB sonopolymer, which can also interact with the CNT walls through non-covalent and/or covalent interactions [90]. However, DCB sonopolymers (and DCB solvent) do not feature any prominent peaks around 1600 cm^−1^ in their Raman spectra [90], in contrast to FFUR-14. Therefore, a careful monitoring of the G-band spectral region can provide additional information on the possible origin of the CH stretching band. The ability to sort functionalized MWCNTs from not functionalized ones is further demonstrated by comparing the TERS spectrum acquired from CNT-A in position 1 (blue line in Figure 7e,f) with the one of CNT-B in position 2 (orange line in Figure 7e,f). The latter is very similar to the TERS spectrum obtained from bare MWCNT (black line in Figure 7e,f), featuring the same D/G ratio and no visible CH stretching band. Conversely, the TERS spectrum in position 1 presents a clearly identifiable CH stretching band at around 2900 cm^−1^; its D/G ratio has a value lower than the unit due to the strong amplification of the fluorene in-plane mode at 1604 cm^−1^, which overcomes the G band intensity. Consequently, the CH stretching band fingerprint is mostly due to the FFUR-14 polymer rather than to the sonopolymers potentially resulting from the DCB solvent degradation, although a weaker contribution from the latter cannot be ruled out. Very interestingly, in correspondence with the presence of polymer on the CNT, we also noticed systematic downshifts of both D and 2D band positions with respect to the TERS spectrum of the bare MWCNT, amounting up to 26 ± 2 cm^−1^ and 28 ± 2 cm^−1^ respectively. This is consistent with Figure 7c, where the downshift regions match with the higher intensity ones in Figure 7b. However, these observations are in disagreement with the conventional Raman characterization reported in Figure 7h,i where the f-MWCNTs (red line) shows slight upshifts of both D and 2D bands, amounting to 9 ± 2 cm^−1^ and 16 ± 2 cm^−1^, respectively (compared to bare MWCNTs; green line). This upshift effect is generally associated with the disentanglement of the nanotube bundles, with a consequent penetration of the polymer within the bundles [61,71,91], resulting in pressure being exerted upon the CNTs [91,92,93]. In contrast to conventional Raman, TERS spectroscopy provides local characterization on portions of individual MWCNTs, allowing a specific analysis of only the surface and the external CNT walls [77]. Therefore, the TERS technique offers a closer look upon the interactions between the functionalizing agent and the external walls of the CNT, unlike the far-field Raman response, which is instead dominated by the global effect of the polymeric matrix acting on the MWCNTs. On the other hand, as already seen in Section 3.3, the shift of 2D peak can be attributed to local strain effects. Notably, when the 2D mode shifts to lower frequencies, the CNT is under tensile stress [84,94,95]. According to the orientation of CNT-B at position 2, it should be under tensile stress, but the 2D band shift is not compatible with such a picture, especially if compared with position 1 in which the CNT-A should present a lower local stress. Finally, we have compared two close positions on the same CNT, i.e., position 3 and 4 (black and red curves in Figure 7g), separated by only 15 nm. They show a relative shift of about 23 cm^−1^, which cannot be related to strain effects, since there is no bending in this region.

Further, the downshift effect observed in TERS is compatible with a change in the local environment because of the presence of the polymeric segments coating the CNT. In fact, if the polymer aromatic groups are actually interacting with the MWCNT carbon lattice, either covalently or most likely by π−π bonding, the electronic distribution within the CNT band structure can be affected by small changes due to charge transfer processes [70,96]. This can possibly induce a shift in the D and 2D band positions [97,98,99], since these two modes are strongly dependent on the carrier density [96] and because of the very efficient electron–phonon coupling [76,100]. Notably, the presence of a prominent downshift, together with the evidence of the polymer bands (both at 1604 and 2900 cm^−1^), suggests that the functionalization process is actually occurring and is very likely acting as an electron doping effect, with the polymer providing additional electrons to the π band of MWCNTs [96], as also suggested by other studies on nanocomposite systems made of polyfluorene wrapped MWCNTs [99].

## 4. Conclusions

In summary, in this work we analyzed the TERS performance of AFM–TERS and TF–TERS at 633 nm using Ag coated AFM tips and Au bulk tips, respectively. We made the comparison on a sample of MWCNTs cast on a flat gold substrate, exploiting the additional field enhancement provided by the formation of a cavity mode between the tip and the substrate. In both TERS modes, the EF values obtained are of the order of 10^4^ to 10^5^. The contrast values range from 10 to 40, indicating negligible far-field contribution and allowing for fast chemical imaging at the nanoscale, with spatial resolution as high as 20 nm—better than the one obtainable with the SPM technique alone, using the same tips—and a chemical sensitivity lower than 10 nm at this excitation wavelength. In addition to its imaging capability, the TERS technique, operated in both modes, has proven to be highly informative due to the valuable structural information of the sample contained in the vibrational spectra. For instance, it is potentially capable of providing the spatial distribution of the local stress, as well as the structural point defects. The comparable results obtained in AFM–TERS and TF–TERS confirm that the initial gap between coated and bulk plasmonic tips is nearly filled. This is due to the strong effort of the scientific community and companies toward the optimization of TERS tips fabrication, which was boosted by the high demand for characterization techniques sensitive to the nanoscale in many application fields. Finally, in the second part of the paper, we demonstrated the applicability of AFM–TERS to the interfacial study of polymer/MWCNT nanocomposites. Notably, by analyzing the surface distribution of both the polymeric component and MWCNTs, we were able sort the CNTs, which are, in fact, covered by polymer from the nearly bare ones in an area of about 200 × 130 nm^2^. Moreover, the shifts of the D and 2D bands of the MWCNT, observed concurrently with evidenced presence of the polymer, suggest that the two components are effectively cooperating through π−π interactions, possibly inducing a rearrangement of the electronic band structure of the MWCNT. Very likely, the polymeric aromatic groups act as an effective electron dopant, causing a downshift in the D and 2D band positions. We have thus proved that AFM–TERS can be successfully used to monitor the quality of the functionalization process of MWCNTs by polymeric derivatives. Further investigations should allow a more precise determination of the origin of these aromatic groups, by sorting the ones related to the FFUR-14 polymer from those associated with the possible sonopolymers originating from the solvent.

## Data Availability

Data are available upon reasonable request from the corresponding authors.

## Supplementary Materials

The following supporting information can be downloaded at: https://www.mdpi.com/article/10.3390/nano12030451/s1, Figure S1: Tips morphology.
