# Supplementary material for: Comparing Commercial Metal-Coated AFM Tips and Home-Made Bulk Gold Tips for Tip-Enhanced Raman Spectroscopy of Polymer Functionalized Multiwalled Carbon Nanotubes
Source: Nanomaterials (Basel). 2022 Jan 28;12(3):451. doi: 10.3390/nano12030451 (PMC8840094; doi:10.3390/nano12030451)
Supplement: Supplementary file 1 [file nanomaterials-12-00451-s001.zip › nanomaterials-1511526-supplementary.pdf]

Supplementary Materials

Comparing Commercial Metal-Coated AFM Tips and Home-Made Bulk Gold Tips for Tip-Enhanced Raman Spectroscopy of Polymer Functionalized Multiwalled Carbon Nanotubes

Antonino Foti [1,2,*], Suriya Venkatesan [2,†], Bérengère Lebental [2,3], Gaël Zucchi [2] and Razvigor Ossikovski [2,*]

1 CNR—IPCF, Istituto per I Processi Chimico-Fisici, Viale F. Stagno d'Alcontres 37, 98158 Messina, Italy
2 LPICM, CNRS, Ecole Polytechnique, Institut Polytechnique de Paris, Route de Saclay, 91128 Palaiseau, France; suriya.venkatesan@dlr.de (S.V.); berengere.lebental@univ-eiffel.fr (B.L.); gael.zucchi@polytechnique.edu (G.Z.)
3 COSYS-LISIS, Université Gustave Eiffel, IFSTTAR, 77454 Marne-la-Vallée, France
* Correspondence: antonino.foti@cnr.it (A.F.); razvigor.ossikovski@polytechnique.edu (R.O.)
† Current address: Department of Electrochemical Energy Technology, Institute of Engineering Thermodynamics, German Aerospace Centre (DLR), D-70569 Stuttgart, Germany.

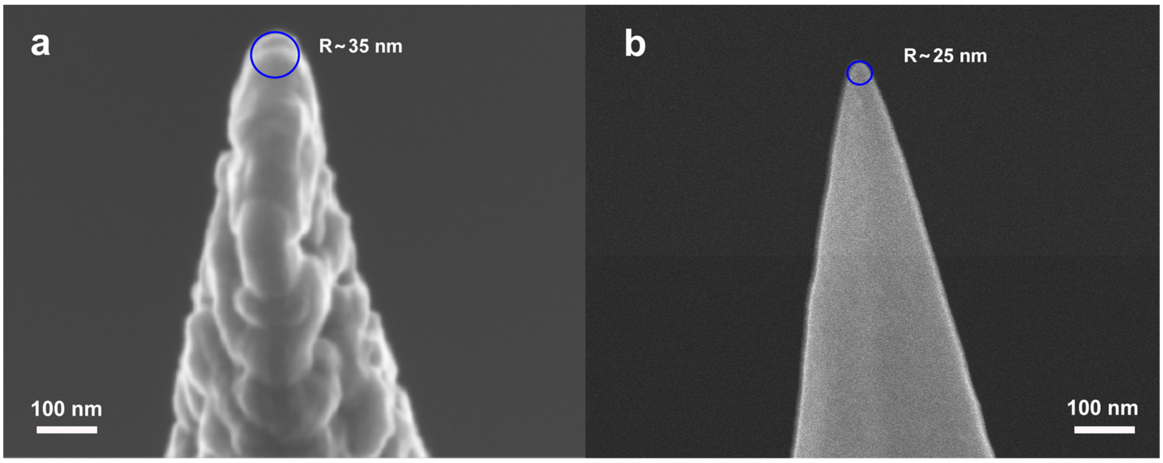

Figure S1. Tips morphology. (**a**) Typical SEM image of a commercial AFM-TERS Tips (Courtesy of Dr. Agnes Tempez from HORIBA Scientific). (**b**) Typical SEM image of an electrochemically etched Au tip.
